# First steps toward a questionnaire of motivational awareness: preliminary development and psychometric examination

**DOI:** 10.3389/fpsyg.2026.1869033

**Published:** 2026-08-31

**Authors:** Lisa Otte, Stefan Fries

**Affiliations:** Department of Psychology, Bielefeld University, Bielefeld, Germany

**Keywords:** experience sampling method, higher education, metamotivation, motivational awareness, motivational conflict

## Abstract

Across two studies, we conducted a preliminary psychometric examination of the newly developed Motivational Awareness Questionnaire (MAQ). Motivational awareness is the ability to consciously differentiate, verbalize, and analyze one’s own motivational states and use this knowledge for deliberate motivation and behavior regulation. Study 1 evaluated the initial MAQ in 142 students. The proposed five-factor structure was tested and not confirmed, necessitating exploratory revisions. Study 2 (*N* = 102) reassessed a revised three-factor version and included ambulatory assessment data capturing motivational states. Although the Bayesian confirmatory factor analysis did not support the proposed three-factor structure (poor model fit), the subscales demonstrated satisfactory internal consistencies. Expected associations with emotional awareness and mindfulness provided initial evidence of convergent associations. Higher differentiation of motivation was associated with less motivational interference and with lower levels of negative affect. In conclusion, the MAQ represents a promising first attempt at measuring motivational awareness, but future research with larger samples is needed to refine the factor structure and establish its validity.

## 1 Introduction

Motivational problems are pervasive in the everyday lives of many students. Many face the challenge of motivating themselves to study, deciding between several possible courses of action, or maintaining their motivation over time. This requires a high degree of self-regulation. Recent studies have additionally focused on the processes through which individuals monitor and control their motivational states in order to attain their goals. These processes are called metamotivation (Miele et al., 2020). These authors conceptualize two interrelated processes: metamotivational monitoring and metamotivational control. The effectiveness of these monitoring and control processes partly depends on the metamotivational knowledge that individuals possess, which aligns with metacognitive frameworks (see Flavell, 1979; Miele and Scholer, 2018; Pintrich, 2002; Wolters, 2003). For example, a student working on a term paper must be aware of both the quantity and quality of motivation required for the task (task knowledge) and of the strategies available to maintain motivation (strategy knowledge). Moreover, knowledge of one’s current motivational state and of which strategy is likely to be most effective (self-knowledge) is essential.

Miele and Scholer (2018) propose that metamotivational knowledge can influence both the monitoring and controlling of motivation, operating at explicit and implicit levels. There may be interindividual differences in the extent of such knowledge. For some students, it might be more difficult to know which strategy to apply to increase their motivation; others may possess the relevant knowledge but struggle to recognize when a particular strategy is most effective. Previous studies have identified associations between metamotivational knowledge and constructs such as motivation, engagement, academic achievement, or psychological well-being (e.g., MacGregor et al., 2017; Ross et al., 2023). In a recent study, Yu et al. (2023) found that metamotivational knowledge can be regarded as an intraindividual resource for academic self-determination. However, research focusing specifically on students’ metamotivational self-awareness remains scarce. Unlike research on metamotivational *knowledge*, which refers to static cognitive representations, the current study aims to address this gap by examining how the capacity to recognize and analyze one’s own motivational states can be assessed.

### 1.1 Motivation and self-regulation

Many learners report a lack of motivation to engage in learning processes (Zimmerman, 2000). However, motivation is a crucial component of learning (Ryan and Deci, 2020; Schunk et al., 2014; Winne, 2001; Zimmerman, 2000). To explain motivation, researchers typically consider, on the one hand, personal characteristics such as personality (e.g., McGeown et al., 2014), fundamental beliefs about one’s own abilities related to the learning task (e.g., Freiberger et al., 2012), as well as interest, motives, motivational orientation, and self-regulation skills (Rheinberg and Fries, 1998). On the other hand, situational characteristics also play a role: for example, task value and expectancy of success are decisive in expectancy-value models (Hulleman et al., 2016) for determining whether motivation is high or low (Rheinberg and Fries, 1998; Wigfield and Eccles, 2000). A general overview of different motivation theories in Educational Psychology can be found, among others, in Eccles and Wigfield (2002) or Wigfield and Koenka (2020).

Although there is extensive evidence on the various factors influencing students’ motivation, there is still limited knowledge about how students actively regulate their own motivation (Miele et al., 2020). In the context of self-regulated learning, the self-regulation of motivation plays a particularly important role (Wolters, 2011). According to Wolters (2003), learners consciously attempt to maintain or enhance their motivation. Both cognitive and behavioral strategies are essential in this process (Wolters, 1998). Based on these considerations, numerous instruments have been developed to capture different motivational regulation strategies used by learners (Schwinger and Otterpohl, 2017; Wang, 2019; Wolters, 1998).

### 1.2 Metamotivation and metamotivational knowledge

A new approach that integrates and extends existing motivation theories is the concept of metamotivation (Miele and Scholer, 2018). This approach is based on the assumption that individuals can monitor both the quantity and quality of their motivation (Miele et al., 2020). The term quantity refers to the extent to which a person wishes to engage in certain activities or tasks - that is, how individuals recognize when their desire to pursue an activity diminishes or increases. In contrast, quality refers to the type or nature of motivational states (e.g., intrinsic vs. extrinsic motivation, promotion focus vs. prevention focus; Miele and Wigfield, 2014).

According to the metamotivation approach, three different types of metamotivational knowledge are considered necessary for regulating one’s own motivation: task knowledge (knowledge about the type and level of motivation required for the task), strategy knowledge (knowledge about the use of strategies to increase motivation levels or to reorient oneself) and self-knowledge (insight into one’s own motivational states and tendencies; Miele and Scholer, 2018). Self-knowledge, as a type of metamotivational knowledge, has hardly been investigated in previous research. Most studies have primarily focused on the fit between task, strategy, and motivational state, while participants’ metamotivational knowledge has typically been assessed indirectly (e.g., through scenario-based studies followed by evaluations of strategies and beliefs; see Scholer and Miele, 2016). In addition to the aspects of task and strategy knowledge, self-knowledge plays a significant role in regulating one’s motivation. Miele and Scholer (2018) have suggested that people can monitor and regulate their motivation by, among other things, observing their metamotivational feelings. For instance, perceiving feelings such as hope or excitement (and the associated thoughts and behaviors) may indicate a promotion-focused motivation. The researchers suggest that people can differ substantially in terms of their self-awareness and emphasize the need for further research to identify predictors of individual or temporal variation in this form of self-knowledge (Miele and Scholer, 2018). For example, it could be assumed that the perception of one’s own mood or the perception of one’s own needs also relates to the perception of one’s own motivational states. These states could be differentiated, for example, into extrinsic vs. intrinsic motivation, high-level construct (abstract and essential) vs. low-level construct (specific and concrete) or promotion versus prevention focus (Miele et al., 2020).

However, the following questions are crucial for advancing research on metamotivation and highlight existing gaps in the literature: Do people generally have access to their motivational state? Can they describe how this state feels subjectively? And what clues help people recognize their current motivational state?

Initial findings on interindividual differences in perceptions of internal states are provided by research on interoceptive perception (Critchley and Garfinkel, 2017) and mood perception (Swinkels and Giuliano, 1995). In 2007, a team of researchers developed a questionnaire that measures children’s and adolescents’ awareness of their own emotions (Rieffe et al., 2007). The Emotional Awareness Questionnaire (EAQ) is an established instrument for measuring emotional awareness. It has been revised and translated into many languages (e.g., Rieffe et al., 2008; Rueth et al., 2019). The EAQ uses six subscales: differentiating emotions, verbal sharing, not hiding emotions, bodily unawareness, attending to others’ emotions, and analyzing (own) emotions. Since emotions and motivation both represent internal control mechanisms for behavior (e.g., Elliot and Thrash, 2002; Harmon-Jones et al., 2012) and represent closely interwoven regulatory systems (e.g., Baumeister et al., 2007; Deci and Ryan, 2000; Koole, 2009), it seems plausible that similar conscious processes–such as recognizing, describing and analyzing inner states - are central in metamotivation. Following the conceptualization of emotion awareness (e.g., Rieffe et al., 2007), which involves attending to emotions to enable adaptive regulation, motivational awareness can be understood as the capacity to attend to motivational states, thereby interrupting automatic behavioral patterns and allowing for more deliberate control. Motivational awareness is conceptually distinct from related metacognitive and self-regulatory constructs such as metamotivational knowledge, control or monitoring as well as self-insight. While knowledge refers to cognitive representations such as task, strategy, and self-knowledge (Miele and Scholer, 2018), awareness focuses on the immediate perception of motivational states. Furthermore, while metamotivational monitoring describes the ongoing process of surveillance and metamotivational controlling refers to the active regulatory actions taken to adjust motivation (Miele and Scholer, 2018), motivational awareness may be viewed as a necessary prerequisite for these processes; it is thought to provide the perceptual information required before monitoring or controlling can occur. Finally, whereas self-insight represents a broad and general form of self-awareness, motivational awareness may be understood as a specific domain of self-awareness, focused on the motivational subsystem.

To develop an instrument to assess the self-reported dispositional levels of motivational awareness, two main challenges need to be considered: (1) identifying the relevant subscales and (2) determining suitable external criteria for initial scale development. The first challenge concerns the identification of subscales that adequately capture different facets of motivational awareness. The theoretical and methodological framework of emotional awareness can serve as a starting point for developing a new instrument to assess motivational awareness. The question arises as to whether and to what extent the structure of the Emotional Awareness Questionnaire can be used as a blueprint for a measure for assessing motivational awareness.

The subscales of the measure to be developed are derived from the following components of emotional awareness: (1) *differentiating motivation*, which refers to the ability to distinguish between different motivational states (e.g., intrinsic vs. extrinsic); (2) *verbal sharing of motivation*, which reflects the capacity to articulate one’s motivational experiences; (3) *bodily awareness of motivation*, which refers to the bodily feeling and physical sensation associated with one’s motivational experiences; (4) *analyzing one’s own motivation*, which captures reflective processes about the causes and consequences of one’s motivational states, and (5) *attending to others’ motivation*, which is defined as the capacity to identify and understand the motivational state of another person.

The second challenge concerns determining suitable external criteria for initial scale development. Metamotivation shares a conceptual overlap with self-regulated learning, as both involve monitoring and control of internal states (Miele and Scholer, 2018; Zimmerman, 2002). Moreover, self-regulated learning is associated with metacognitive knowledge, strategy repertoire, and feedback monitoring (Bürgler et al., 2021). In both frameworks, learners consciously observe their own thoughts, emotions, and motivational tendencies in order to adapt their behavior to situational demands. Such processes require awareness of one’s motivational dynamics and the ability to deliberately regulate them in line with personal goals. Consequently, awareness of one’s motivational processes may be conceptualized as a potential contributing factor for adaptive self-regulation in academic contexts.

It can therefore be assumed that students with higher motivational awareness also demonstrate stronger self-regulatory abilities, greater well-being, and, ultimately, higher academic functioning. Life satisfaction represents a central indicator of subjective well-being and is closely related to adaptive self-regulation (Deci and Ryan, 2000; Ryan and Deci, 2020) and motivational competence (Grund et al., 2018). Individuals who are more aware of their motivational dynamics are better able to align their goals with their personal needs and values, which is likely to contribute to greater life satisfaction. Previous studies have shown that students who can effectively monitor and regulate their motivation tend to be more engaged and achieve better academic outcomes (Schwinger and Otterpohl, 2017; Wolters, 2011). Accordingly, motivational awareness should be positively associated with academic achievement. The ability to control motivation, attention, and mood reflects core aspects of self-regulation and executive functioning (Fujita et al., 2024b; Zimmerman, 2002). Since metamotivational processes involve the monitoring and regulation of internal states, individuals with higher motivational awareness are expected to report a stronger perceived sense of control in these domains.

Another important aspect in the context of motivational awareness concerns the potential relationship between mindfulness and motivational awareness. Mindfulness should help individuals remain functional, efficient, and psychologically well-adjusted in motivationally challenging situations (Grund et al., 2021). An increased awareness of one’s own motivational states may also be related to a more differentiated and realistic academic self-concept, as both constructs involve self-related evaluative processes. Finally, motivational conflicts and the tendency to experience motivational interference between study and leisure activities reflect the dynamic interplay of competing goals (Grund et al., 2015). Students with higher motivational awareness are likely to recognize such conflicts earlier and to employ adaptive strategies to reduce interference.

### 1.3 Objectives of the present study and hypotheses

To better assess motivational awareness, it is essential to develop appropriate measurement tools. A questionnaire designed to assess the self-reported dispositional levels of motivational awareness represents an approach to capturing individuals’ ability to understand and analyze their own motivational processes. In the present paper, we report on the preliminary development of a questionnaire, aiming to address a notable gap in the field.

Two datasets were analyzed to test the hypotheses described below. The first dataset stems from a pilot study in which students answered an online survey. The second study contains data from a larger ambulatory assessment study, which was preregistered on the Open Science Framework (OSF; https://osf.io/2wteu). Although this larger project collected a wider range of variables (e.g., physical activity), only the motivation-related measures are examined in the present investigation.

The first study aimed to preliminarily examine the factor structure and to gather initial evidence of validity for the newly developed Motivational Awareness Questionnaire (MAQ). This instrument was designed to measure individual differences in self-reported dispositional levels of motivational awareness. Specifically, this study investigated whether the MAQ reflects the assumed five-factor structure, comprising the following subscales outlined above: (1) *differentiating motivation*, (2) *verbal sharing of motivation*, (3) *bodily awareness of motivation*, (4) *attending to others’ motivation*, (5) *analyzing one’s own motivation*. Furthermore, we addressed the following research questions: Do the individual subscales of the MAQ demonstrate acceptable internal consistency (as measured by Cronbach’s α)? Does the MAQ correlate with related external constructs in expected directions, thereby providing preliminary evidence of convergent associations?

Hypothesis 1: The assumed five factors of the Motivational Awareness Questionnaire (MAQ) comprising the *subscales* (*differentiating motivation, verbal sharing of motivation, bodily awareness of motivation*, *attending to others’ motivation*, and *analyzing one’s own motivation*) can be empirically confirmed.

Hypothesis 2: The internal consistencies of the individual subscales of the questionnaire are satisfactory (indicated by alpha).

Hypothesis 3: Higher scores on the MAQ subscales are positively related to adaptive constructs (specifically, life satisfaction, academic success, mindfulness, academic self-concept, and the general ability to control motivation, attention, and mood) and negatively related to maladaptive or conflict-related constructs (specifically, Motivational Conflicts and the Tendency to Experience Motivational Interference in Study and Leisure).

## 2 Study 1: pilot study of a questionnaire to assess students’ motivational awareness

### 2.1 Method

#### 2.1.1 Participants

In this study, 149 students filled out an online questionnaire. The data of seven participants were excluded from the analyses because they were not enrolled students. Participants were rewarded with course credits or the chance of winning one of five €30 vouchers. The sample used for data analyses consisted of 142 students, including 21 males and 121 females. Recruitment was carried out via a survey system of our university, survey networks and social networks. The participants’ age ranged between 18 and 48 years (*M* = 23.46; *SD* = 4.93). Of the participants, 64.1% studied psychology. The average university semester was 4.14 (*SD* = 3.67). 73.9% of the participants were aiming for a Bachelor’s degree at that time. 64.8% of the participants stated that they had another commitment in addition to their studies [part-time job (53.5%), other commitments (9.2%), own children (1.4%), caring for relatives (0.7%)].

#### 2.1.2 Instruments

##### 2.1.2.1 Motivational awareness

The Motivational Awareness Questionnaire aims to identify the students’ access to their own motivation. The items of the MAQ were developed based on the Emotion Awareness Questionnaire (EAQ). To ensure their clarity and comprehensibility, the items were pre-tested in a small pilot sample of 30 students. Furthermore, the questionnaire was reviewed for comprehensibility and face validity by a student focus group and two experts in motivational psychology. Based on their feedback, minor adjustments were made to the phrasing before administering the questionnaire in the main studies. The MAQ (28-items) was originally designed with a five-factor structure and administered in German. The English items presented in this manuscript (see Appendix A) are translations of the original German items. To ensure semantic equivalence and clarity, the translation process was conducted using a forward-backward translation procedure by two independent translators. The complete list of the original German items is provided in Appendix B.

The first factor, *differentiating motivation,* reflects the ability to distinguish between various motivational states, such as recognizing differences in intensity or type of motivation (for example, intrinsic versus extrinsic motivation; example item: “*I am often confused or at a loss as to whether I am motivated or unmotivated.”*). The second factor, *verbal sharing of motivation*, refers to the tendency and capacity to express one’s motivational experiences verbally to others; thereby, self-reflection is needed (example item: *“I can easily explain to a friend how I motivate myself.”*). The third factor, *bodily awareness of motivation*, captures the extent to which individuals perceive bodily sensations or physical cues associated with their motivational states, such as restlessness, energy, or tension (example item*: “When I am unmotivated, my body feels exhausted.”*). The fourth factor, *attending to others’ motivation*, describes the awareness of other people’s motivational states and the ability to interpret behavioral or emotional cues that indicate whether others are engaged or demotivated (example item: “*I can usually tell whether my friends are motivated or unmotivated.”*). Finally, the fifth factor, *analyzing one’s own motivation*, represents the reflective component of motivational awareness – the tendency to analyze what drives one’s motivation, why it changes, and how it might be improved (example item*: “When I am unmotivated, I try to understand why.”*). Respondents were asked to rate the degree to which each statement applies to them on a six-point scale (1 = not true at all, 2 = not true, 3 = rather not true, 4 = rather true, 5 = true, 6 = exactly true).

##### 2.1.2.2 Life satisfaction

Life satisfaction was assessed by the Temporal Facets of Life Satisfaction (Trautwein, 2004) using four items. The factor structure was examined in the German validation study (Trautwein, 2004; *N* = 3,183) using confirmatory factor analysis. A three-factor model with correlated item residuals showed good fit, RMSEA = 0.06, TLI = 0.96, and clearly outperformed an uncorrelated three-factor model (RMSEA = 0.08, TLI = 0.92) as well as a one-factor model (RMSEA = 0.48). Internal consistency of the subscales ranged between *α* = 0.80 and *α* = 0.90. Various statements such as “I am satisfied with my current life” were rated on a four-point Likert scale from 1 (strongly disagree) to 4 (strongly agree). The internal consistency and other descriptives are shown in Appendix C.

##### 2.1.2.3 Perceived academic success

Perceived academic success was assessed using a single self-developed item. Participants were asked to rate their academic performance relative to their fellow students (“How would you rate your academic success compared to your fellow students?”). Responses were given on a 5-point scale ranging from 1 (well below average) to 5 (well above average), with 3 indicating average performance.

##### 2.1.2.4 Ability to control motivation, attention, and mood management

Self-regulatory abilities were assessed with the motivation control, attention control, and mood management subscales of the Self-Control Inventory (SSI; Kuhl and Fuhrmann, 1998). To our knowledge, no published confirmatory factor analysis exists for the SSI; its factor structure has so far only been examined exploratorily (principal component analysis). We therefore report here the published reliabilities (Motivation Control: *α* = 0.90; Attention Control: *α* = 0.81; mood management: *α* = 0.86) as evidence of the psychometric quality of the subscales used. The 15 items are rated using a four-point Likert scale from 1 (does not apply at all) to 4 (applies completely). An example item is “When something gets boring, I usually know how to make it fun again.” The internal consistency and other descriptives are shown in Appendix C.

##### 2.1.2.5 Mindfulness

Mindfulness was measured with the German version of the Kentucky Inventory of Mindfulness Skills (KIMS-D; Ströhle et al., 2010), which comprises 39 items rated on a 5-point Likert scale ranging from 1 (never or very rarely true) to 5 (very often or always true). An example item is “It is easy for me to put my beliefs, opinions and expectations into words.” The scoring is reversed for 17 items. The factor structure was confirmed in the German validation study (Ströhle et al., 2010; *N* = 469) using confirmatory factor analysis. A correlated-factors model showed acceptable fit (RMSEA = 0.059, CFI = 0.97), and a second-order model with a superordinate mindfulness factor likewise showed acceptable, though somewhat weaker, fit (RMSEA = 0.066, CFI = 0.96; *χ*^2^ difference test significant in favor of the correlated-factors model). Internal consistency of the four subscales ranged between *α* = 0.79 to *α* = 0.92. The internal consistency and other descriptives from our study are shown in Appendix C.

##### 2.1.2.6 Academic self-concept

Academic self-concept was measured by 5 self-generated items, which are rated on a six-point Likert scale from 1 (not true at all) to 6 (exactly true). The items are: “Even though I try hard, I think studying is difficult.”; “I simply have no talent for my studies.”; “I don’t particularly enjoy my studies.”; “I think my studies are difficult.”; “With some things in my studies I know straight away: I’ll never understand this!” All items were reverse-coded so that higher values indicate a more favorable academic self-concept. The internal consistency and other descriptives are shown in Appendix C.

##### 2.1.2.7 Motivational conflicts

The frequency of experiencing motivational conflicts was measured after a short explanation about motivational conflicts by the self-generated item “How often do you experience such or similar conflicts between your studies and your free time?”. The answers were 1 (never); 2 (rarely); 3 (sometimes); 4 (often); 5 (very often). The internal consistency and other descriptives are shown in Appendix C.

##### 2.1.2.8 Tendency to experience motivational interference

The tendency to experience motivational interference was assessed using 22 items (Grund and Fries, 2014). Twelve items were assigned to the scale *Tendency to Experience Motivational Interference during Studies (TMI-S)* and 10 items to the scale *Tendency to Experience Motivational Interference during Leisure Time*. The factor structure was examined by Grund and Fries (2014) using confirmatory factor analysis. For both scales, a second-order structure with four subfacets and one superordinate conflict score was confirmed: TMI-S showed good fit, *χ^2^*(50) = 127.61, *p* < 0.001, CFI = 0.95, RMSEA = 0.08, SRMR = 0.05; TMI-L showed acceptable fit, *χ^2^*(31) = 116.79, *p* < 0.001, CFI = 0.93, RMSEA = 0.11, SRMR = 0.07. The respondents were asked to use a five-point Likert scale from 1 (rarely) to 5 (almost always) to indicate the frequency with which they generally experience this feeling when they are either studying or pursuing a leisure activity. An example item is “How often do you feel annoyed in such situations because you could make better use of your time?”. The internal consistency and other descriptives are shown in Appendix C.

#### 2.1.3 Procedure

The data of the online survey study were collected using the *Qualtrics* survey tool. Before the start date of the survey, the study was approved by an Ethics Committee of Bielefeld University (2025-043). The study was preregistered under https://aspredicted.org/7xzp-3yhh.pdf. All deviations from the preregistered analysis plans are transparently reported below and summarized in Appendix D. After the participants answered a few questions about their demographics, the individual instruments mentioned above were presented one after another.

#### 2.1.4 Data analysis

The statistical analyses used to test the hypotheses were based on a sample size of *N* = 142 and were carried out using the IBM SPSS computer program (Version 31.0, 2025). First, to test the proposed five-factor structure of the MAQ, a confirmatory factor analysis (CFA) was conducted through Mplus (version 8; Muthén and Muthén, 1998–2017). The assessment of the fit indices is based on the criteria of Hu and Bentler (1999), who postulate a satisfactory goodness of fit if the comparative fit values (CFI/TLI) are above a value of 0.90 or 0.95, the root mean square error of approximation (RMSEA) is below a value of 0.06, and the standardized root mean square residual (SRMR) ≤ 0.08. Missing data were coded as −99 and treated as missing values in all analyses. Items were first examined for univariate normality, revealing eight items (1.4, 1.6, 1.7, 3.1, 3.2, 3.3, 4.4, 5.2) with pronounced negative (left) skewness. None of the mentioned items scored above the threshold (skewness = 2, kurtosis = 7; Curran et al., 1996), but their critical ratios were above 1.96. Hence, we must assume that the skewness or kurtosis, based on a significance level of *p* < 0.05, significantly deviates from zero. Because multivariate normality could not be achieved, the robust maximum likelihood estimator (MLR) was used for model estimation. This estimator produces scaled chi-square values and is robust to nonnormality (Muthén and Muthén, 1998–2017).

### 2.2 Results of Study 1

#### 2.2.1 Descriptive statistics

Means, standard deviations, and internal consistencies (Cronbach’s *α*) of the five subscales are presented in Table 1. Internal consistencies of the MAQ subscales ranged from *α* = 0.70 (*attending to others’ motivation*) to *α* = 0.84 (*differentiating motivation*), while descriptive statistics for all other study variables are reported in Appendix D.

**Table 1:** Means, standard deviation and internal consistencies of the MAQ-28.

Subscales	Items	*M*	SD	*α*	95% CI	*ω*	Inter-item correlation
Differentiating motivation	9	4.10	0.75	0.84	[0.80, 0.88]	0.85	0.38
Verbal sharing of motivation	4	4.07	0.92	0.78	[0.72, 0.84]	0.77	0.48
Bodily awareness of motivation	4	4.82	0.76	0.77	[0.71, 0.83]	0.74	0.46
Attending to others’ motivation	4	4.29	0.81	0.70	[0.61, 0.77]	0.68	0.35
Analyzing one’s own motivation	7	4.05	0.78	0.82	[0.77, 0.86]	0.78	0.39

MAQ-28 = Motivational Awareness Questionnaire, Items = Number of items, α = Cronbach’s alpha, CI = confidence interval, ω = McDonald’s omega.

#### 2.2.2 Psychometric properties of the MAQ scores

The intercorrelations among the MAQ-28 subscales were generally positive and ranged from small to strong (see Table 2).

**Table 2:** The interfactor correlations of the MAQ-28.

Subscales of the MAQ	Verbal sharing of motivation	Bodily awareness of motivation	Attending to others’ motivation	Analyzing one’s own motivation
Differentiating motivation	0.66**	−0.09	0.12	0.32**
Verbal sharing of motivation		−0.04	0.23**	0.42**
Bodily awareness of motivation			0.27**	0.16
Attending to others’ motivation				0.36**

*N* = 142. **p < 0.01 (two-tailed).

##### 2.2.2.1 Confirmatory factor analysis

The results of the confirmatory factor analysis (CFA) did not support the assumed five-factor structure of the MAQ: *χ^2^* (340) = 637.68, *p* < 0.001, CFI = 0.81, TLI = 0.78; RMSEA = 0.079 (90% CI [0.069, 0.088]), SRMR = 0.100, AIC = 10925.981; BIC = 11203.829. Moreover, the modification indices indicated that items 5.6 and 1.6, as well as several items from the subscales *bodily awareness of motivation* and *attending to others’ motivation*, showed highly correlated error terms.

#### 2.2.3 Convergent associations

The correlation coefficients (Table 3) show that all scales showed substantial and significant relations to one or more dependent variables.

**Table 3:** Correlation coefficients for MAQ-28 scales and relevant constructs.

Questionnaire	Differentiating motivation	Verbal sharing of motivation	Bodily awareness of motivation	Attending to others’ motivation	Analyzing one’s own motivation
Correlations					
TFL	0.38**	0.35**	0.03	−0.01	0.24**
Academic success	0.18*	0.17*	−0.11	0.04	0.14
SSI					
SSI–Motivation control	0.37**	0.35**	0.09	0.30**	0.24**
SSI- Attention control	0.47**	0.29**	−0.12	0.21	0.32**
SSI - Mood management	0.23**	0.17*	0.14	0.19*	0.22**
KIMS-D					
KIMS-D observation	0.13	0.26**	0.29**	0.25**	0.43**
KIMS-D attention	0.43**	0.23**	−0.21*	−0.00	0.25**
KIMS-D describing	0.43**	0.51**	0.00	0.11	0.37**
KIMS-D accepting	0.32**	0.29**	−0.20*	−0.08	−0.05
Academic self-concept	0.29**	0.35**	−0.13	−0.03	0.21*
TMI					
TMI-S	−0.45**	−0.28**	0.14	−0.01	−0.33**
TMI-L	−0.23**	−0.21*	0.21*	−0.03	−0.08
Motivational conflicts	−0.21*	−0.08	0.28**	0.07	−0.03

N = 142. TFL = Temporal Facets of Life Satisfaction; SSI = Self-Control Inventory; KIMS-D = Kentucky Inventory of Mindfulness Skills; TMI = Tendency to Experience Motivational Interference during Studies (TMI-S) and during Leisure Time (TMI-L). **p < 0.01. *p < 0.05 (two-tailed).

#### 2.2.4 Exploratory model modification and refinement

Given the poor fit of the initially hypothesized five-factor model, we conducted exploratory, post-hoc modifications using the same dataset to refine the scale. Because these modifications were data-driven and not pre-registered, the resulting model fit must be interpreted strictly as exploratory rather than confirmatory. Exploratory analyses were not corrected for multiple testing and should therefore be interpreted as hypothesis-generating.

First, based on conceptual redundancy and poor initial performance, we excluded two entire subscales: *bodily awareness of motivation* and *attending to others’ motivation*. Second, we removed three items due to questionable factor loadings or content-related mismatch during *post hoc* model refinement in Study 1: Items 1.6 and 1.7 from the *differentiating motivation* subscale and Item 5.6 from *the analyzing one’s own motivation* subscale.

These decisions were made after inspecting model fit and item performance in the original dataset. The specific decision criteria were not preregistered. Therefore, the revised model and its improved fit should be interpreted as exploratory rather than confirmatory.

Following these post-hoc modifications, the remaining items loaded cleanly on their respective factors: *differentiating motivation* (0.503, 0.517, 0.639, 0.465, 0.686, 0.478, 0.643), *verbal sharing of motivation* (866, 0.538, 0.615, 0.611), *analyzing one’s own motivation* (0.807, 0.462, 0.592, 0.895, 0.624, 0.622).

To evaluate the structure of this modified model, we re-ran the analysis on the same dataset. Although the model fit improved substantially, this must be considered a preliminary and exploratory result. A bootstrap procedure was applied because the assumption of normality was not met, *χ^2^* (116) = 186.921, *p* < 0.001, CFI = 0.92, RMSEA = 0.066 [0.048, 0.083], SRMR = 0.066, AIC = 260.921, BIC = 271.750.

Regarding the fit indices, the SRMR indicated a good fit between the data and the model. The RMSEA was only slightly above the desired threshold of 0.06. The CFI was also slightly below but still close to the preferred value of 0.95 and above the previously suggested cutoff of 0.90 (Hu and Bentler, 1999). All standardized factor loadings in this exploratory model were significant, ranging from 0.522 to 0.795, and the latent factor correlations were positive and significant (*r* = 0.32 to 0.66).

### 2.3 Brief discussion

The analyses showed that the model fit is not entirely satisfactory. This could be explained by the very similar content and wording of some items, but also by the fact that the two scales mentioned, *bodily awareness of motivation* and *attending to others’ motivation*, do not represent conceptually distinct facets of motivational awareness. Furthermore, the relatively small sample size may have introduced biases and instability in both parameter estimates and fit indices. Although the post-hoc modifications to the MAQ resulted in a substantially improved model fit, this fit must be interpreted with caution. Because the revised three-factor model was evaluated using the same dataset from which the modifications were derived, the fit indices are exploratory and likely over-optimistic due to capitalization on chance. Based on these substantive considerations and exploratory findings, we concluded that it would be beneficial to further develop the MAQ by restricting it to three subscales (*differentiating motivation*, *verbal sharing of motivation*, and *analyzing one’s own motivation*) and adding new items to strengthen these remaining dimensions. Nonetheless, the initial version of the MAQ demonstrated promising relations with several relevant external criteria, underscoring the value of continuing this line of research.

## 3 Study 2: development of a motivational awareness questionnaire (MAQ)

In this second study, the research questions were further addressed using a new sample and new design features. The questionnaire was tested empirically in its revised version, based on the proposed structure of three subscales (*differentiating motivation*, *verbal sharing of motivation*, *analyzing one’s own motivation*). Compared to Study 1, Study 2 also included data at the situational level, which were collected as part of a large ambulatory assessment study.

To examine the preliminary internal consistency and gather initial validity evidence for the newly developed MAQ, three hypotheses were formulated concerning internal consistency, associations with external criteria on the person level, and associations with external criteria on the situational level. We tested the following preregistered (OSF; doi: 10.17605/OSF.IO/RKV6U) hypotheses:

Hypothesis 1: The proposed three-factor structure of the MAQ, comprising the subscales *differentiating motivation*, *verbal sharing of motivation*, and *analyzing one’s own motivation*, can be empirically confirmed. Furthermore, the internal consistencies of the individual subscales are satisfactory (as indicated by alpha).

Hypothesis 2: At the person level, MAQ subscale scores are positively associated with mindfulness, emotional awareness, and self-control, and negatively associated with the general experience of motivational conflicts and motivational interference.

Hypothesis 3: At the situational level, MAQ subscale scores are positively associated with well-being and perceived learning performance in everyday life, and negatively associated with the experience of motivational conflicts and motivational interference in everyday life.

In addition, we examined whether motivational awareness reflects a relatively stable construct. This question was addressed in an exploratory analysis that was not preregistered.

Exploratory Hypothesis 4: There is a significant positive correlation between the MAQ scores in the pre-, interim-, and post-test, measured at intervals of 1 and 2 weeks.

### 3.1 Method

#### 3.1.1 Participants

Hundred and two students took part in Study 2 (73.5% female; 26.5% male). The students’ age ranged between 18 and 39 (*M* = 23.68; *SD* = 4.94). 54.9% of the participants studied psychology, 18.6% teaching education programs, and 26.5% studied other subjects. The average number of semesters spent at university was 5.20 (*SD* = 3.92).

#### 3.1.2 Instruments

For Study 2, the MAQ used in Study 1 was adapted and reduced to three subscales (1) *differentiating motivation*, (2) *verbal sharing of motivation* and (3) *analyzing one’s own motivation*. Prior to data collection, several items were adapted and reformulated during the scale refinement process. These decisions were made after inspection of the Study 1 data. The revised model is therefore considered exploratory. For the subscale *differentiating motivation*, Items 1.6 and 1.7 were removed, and one new item was developed to better capture the intended construct domain. For the subscale *verbal sharing of motivation* four additional items were developed and included to improve content coverage. Within the subscale *analyzing one’s own motivation*, Items 5.2 and 5.7 were reformulated for clarity, Item 5.6 was removed, and two new items were added. The new adapted version of the MAQ is shown in Table 4 (for German Version see Appendix E). The model refinement in Study 1 was exploratory. The revised version of the MAQ was subsequently tested in Study 2 following a preregistered analysis plan.

**Table 4:** MAQ items (24 items).

Differentiating motivation
1.1 I am often confused or at a loss as to whether I am motivated or unmotivated -R
1.2 I often cannot say what motivates me -R
1.3 I often cannot say why I do or do not do something -R
1.4 I find it difficult to say whether I am motivated or unmotivated. -R
1.5 I often do not know exactly whether I do something because I like doing it or because I feel obliged to. -R
1.6 When I am unmotivated, I do not know what feeling (e.g., fear, boredom, hopelessness) could be responsible. -R
1.7 Sometimes I feel unmotivated and do not know why. -R
1.8 I find it easy to recognize whether I am doing something voluntarily or because others expect me to do it.

Verbal sharing of motivation
2.1 I find it difficult to explain to a friend what motivates me. -R
2.2 I find it difficult to talk to someone about my motivation. -R
2.3 I can easily explain to a friend how I motivate myself.
2.4 I can easily explain to a friend why I am unmotivated.
2.5 I can describe well what motivates me in certain situations.
2.6 I am good at describing my motivational state in an understandable way.
2.7 I sometimes find it difficult to explain to someone why I am motivated or unmotivated. -R
2.8 When I talk about my motivation, I can explain it clearly.

Analyzing one’s own motivation
3.1 When I am unmotivated, I try to understand why.
3.2 The nature of my motivation helps me to understand what I like to do.
3.3 If I have a motivation problem, it helps me to find out whether an emotion (e.g., fear, hopelessness, boredom) could be responsible.
3.4 It is important for me to understand whether I am motivated or unmotivated.
3.5 I always want to know how I can motivate myself.
3.6 When I am unmotivated, I try to analyze how I can motivate myself.
3.7 I often analyze the connections between my emotions and my motivation.
3.8 It is important for me to understand the causes of my motivational state.

Items marked with R are reverse-scored.

The other measuring instruments were nearly the same as in Study 1. Relevant for the present study were the observing, acting with attention, describing, and accepting without judgment subscales of the German version of the Kentucky Inventory of Mindfulness Skills (KIMS-D; Ströhle et al., 2010), the experience of motivational conflicts and motivational interference [Tendency to experience motivational interference (TMI); Grund and Fries, 2014], Self-control Scale (SCS-K-D, Bertrams and Dickhäuser, 2009). The internal consistency and other descriptives of all the constructs are shown in Appendix F.

The following measuring instruments were added beyond those used in Study 1:

##### 3.1.2.1 Emotion awareness

We used the facets of differentiating, verbalizing and analyzing emotions of the EAQ (German Version EAQ, Rueth et al., 2019).

##### 3.1.2.2 Self-control

Unlike initially planned in the preregistered hypotheses, we used the Self-Control Scale (SCS-K-D; Bertrams and Dickhäuser, 2009). The unidimensional structure was confirmed by Bertrams and Dickhäuser (2009; *N* = 303) using confirmatory factor analysis. The single-factor model showed good fit, *χ^2^*(56) = 86.71, *p* < 0.01, RMSEA = 0.04, CFI = 0.95, SRMR = 0.04. Internal consistency of the scale was *α* = 0.78–0.79. This questionnaire measures, across 13 items rated on a five-point Likert scale from 1 (does not apply at all) to 5 (fully applies), the ability to control and regulate oneself. An example item is: “I am good at working toward long-term goals.”

At the situation level, we used the following measuring instruments:

##### 3.1.2.3 Wellbeing

The short scales for recording positive activation, negative activation and valence in experience sampling studies (PANAVA-KS; Schallberger, 2005) were used to record well-being. The construct validity was examined by Schallberger (2005); *N* = 269 using confirmatory factor analysis in comparison with established instruments (PANAS, AD-ACL, MDBF-GS). A three-factor model (Positive Activation, Negative Activation, Valence) showed excellent fit, *χ^2^*(31) = 31.2, *p* = 0.46, CFI = 1.00, RMSEA = 0.01, and outperformed two-factor alternative models. Using nine semantic differentials, the well-being of the participants was recorded at seven measurement points throughout the day. The responses were rated on a scale ranging from −3 to +3. Participants were asked how they felt directly before the alarm (valence: satisfied - dissatisfied, unhappy – happy; positive activation: full of energy - lacking energy, tired - wide awake, listless - highly motivated, enthusiastic - bored; negative activation: stressed - relaxed, peaceful - annoyed, calm - nervous, worried - worry-free).

##### 3.1.2.4 Perceived learning performance

Perceived learning performance was measured using one item. Every evening, the participants rated their perceived daily learning performance during their studies on a scale of 0 to 15 points.

##### 3.1.2.5 Motivational conflicts and motivational interference

At five measurement points during the day, the participants answered the questions as to whether they would rather be doing something else (want conflict) or should be doing something else (should conflict). If there was a conflict, participants were asked when exactly they felt they want/should do something else: 0 (before the start of the current activity (motivational conflict)); 1 (during the current activity (volitional conflict)); 2 (during the enquiry (method-induced conflict)). Motivational interference was measured using five items on mood during the activity, distractibility, thoughts about alternatives, task switching and low perseverance (see Stojanovic et al., 2020). Participants were asked to rate the statements on a Likert scale from 1 (strongly disagree) to 5 (strongly agree).

#### 3.1.3 Procedure

For each participant, data collection lasted 15 days. The present analysis focuses on a subset of the data collected. On the first day, participants filled out a baseline questionnaire. This baseline questionnaire consisted of various questionnaires, not all of which were used for this study. The questionnaires used included the MAQ, the German version of the Kentucky Inventory of Mindfulness Skills (KIMS-D; Ströhle et al., 2010), general experience of motivational conflicts and motivational interference (Tendency to Experience Motivational Interference, TMI; Grund and Fries, 2014), and the facets differentiating, verbalizing, and analyzing emotions from the EAQ (German Version, Rueth et al., 2019). According to the preregistration, we intended to assess participants’ general ability to control motivation, attention, and mood management as an external criterion. However, due to an administrative oversight during survey preparation, the preregistered measure was not implemented. Instead, the Self-Control Scale – Short Version (SCS-K-D; Bertrams and Dickhäuser, 2009) was administered. The SCS-K-D captures a closely related construct, namely the general capacity for self-control and self-regulation, which conceptually overlaps with the intended ability measure.

On the second day, the ambulatory assessment started and lasted a total of 14 days. It included seven short surveys per day, administered via the app movisensXS. In addition, the survey included accelerometry (Actigraph wGT3X-BT) and electrocardiography (Polar H10); however, these data are not relevant to the present study. Furthermore, on days 8 and 14, participants filled out longer questionnaires (i.e., interim and end questionnaires) with the same variables as at the baseline.

As compensation for participating in the study, participants could choose between a €100 voucher or 8 research credits and, if they studied psychology, 8 research credits plus a €60 voucher. A subsample of about 30 participants received an additional €20 voucher because they opted to answer a few additional items that were not included in the regular study. Recruitment took place via email distribution lists, social media, digital flyers and direct approaches. Participants had to be at least 18 years old and enrolled as students at the time of the study. Further prerequisites included being a resident of Germany and having German language skills at a level of at least B2 to sufficiently understand the questionnaires. Data collection was conducted in two batches (Batch 1: 4th May - 18th May 2025, Batch 2: 20th June - 6th July 2025). Study 2 was also approved by the Ethics Committee of Bielefeld University before the start of the survey (2025–108). This study was preregistered on the Open Science Framework (OSF; doi: 10.17605/OSF.IO/RKV6U). All deviations from the preregistered analysis plans are transparently reported and summarized in Appendix D.

#### 3.1.4 Data analysis

The data were processed using IBM SPSS Statistics (Version 31.0; IBM Corp, 2025) for descriptive analyses, correlations, reliability estimates, and repeated-measures ANOVAs; all confirmatory factor analyses and multilevel models were estimated in Mplus (Version 8; Muthén and Muthén, 1998–2017). Each participant completed multiple Experience Sampling Method (ESM) measures over a two-week period. Consequently, the situations (Level 1) are nested within individuals (Level 2), indicating that the observations are not independent. Therefore, the use of multilevel analyses was justified to account for the hierarchical nature of the data.

The confirmatory factor analysis (CFA) was conducted in Mplus (Version 8; Muthén and Muthén, 1998–2017) using Bayesian estimation due to the relatively small sample size (*N* = 102) and model complexity. Bayesian estimation provides more stable parameter estimates, is robust to non-normality, and allows posterior predictive checks for assessing model fit (Geiser, 2011; Muthén and Muthén, 1998–2017). By default, Mplus utilized diffuse (non-informative) prior distributions for all parameters: normal priors with infinite variance [*N* (0, ∞)] for factor loadings and intercepts, inverse-gamma priors [*IG* (−1,0)] for residual variances, and an inverse-Wishart prior [*IW* (0, − 4)] for the latent factor covariance matrix. The estimation was performed using two Markov Chain Monte Carlo (MCMC) chains with a maximum of 100,000 iterations (with thinning set to 10). Convergence was monitored dynamically using the Potential Scale Reduction (PSR) factor, with a PSR value < 1.05 taken as evidence of successful convergence.

To evaluate overall model fit, we performed a posterior predictive check (PPC) yielding a posterior predictive *p*-value (*PPP*) and a 95% confidence interval for the difference between the observed and replicated chi-square values. A *PPP*-value of approximately 0.50 and a 95% confidence interval including zero indicate an excellent model fit (Muthén and Muthén, 1998–2017). Although traditional fit indices like the Comparative Fit Index (CFI), Tucker-Lewis Index (TLI), and Root Mean Square Error of Approximation (RMSEA) were originally developed for frequentist maximum likelihood estimation, we report their Bayesian counterparts (BCFI, BTLI, and BRMSEA) computed in Mplus (Muthén and Muthén, 1998–2017). These indices are presented to facilitate comparison with traditional fit standards, though they should be interpreted with caution as their exact cutoffs under Bayesian estimation have not been fully established (Garnier-Villarreal and Jorgensen, 2020).

Missing data were coded as −99 and treated as missing values in all analyses. In Mplus, missing data were handled via the Bayesian estimation algorithm (under the missing-at-random assumption), whereas missing data in the multilevel analyses in SPSS were handled using full information maximum likelihood (FIML). In addition to the correlation analyses at the trait level, motivational awareness was related to the situational data obtained from the ambulatory assessment.

Specifically, two-level random-intercept models were estimated to examine whether the three MAQ subscales - *differentiating motivation*, *verbal sharing of motivation*, and *analyzing one’s own motivation* - predict the average levels of experienced motivational interference, well-being, and perceived learning performance at the person level. Multilevel models were estimated using maximum likelihood with robust standard errors (MLR). All models converged normally without warnings. The two-level models partitioned variance in the outcome variables into within-person and between-person components. The MAQ subscales were specified as between-person (Level 2) predictors of the person-level mean of the respective daily outcomes. Level 2 predictors were entered in their observed metric; no additional centering procedures were applied. No additional covariates were included.

To examine the associations between the three MAQ trait scales and the experience of motivational action conflicts in daily life, that is, motivational “want” conflicts (MC_W) and motivational “should” conflicts (MC_S), two two-level logistic multilevel models with a logit link function were estimated. The MAQ scales were specified as Level 2 predictors, whereas the dichotomous ESM variables - motivational “want” conflicts and motivational “should” conflicts - were modeled as Level 1 outcomes.

Standardized regression coefficients were calculated for all linear models. The number of available observations varied depending on the combination of variables included in each model. Participants were prompted seven times per day over 14 days, resulting in 9,996 scheduled assessments. Of these, 7,688 assessments were completed, corresponding to an overall compliance rate of 76.9%. For survey-level analyses, well-being was assessed at all seven daily surveys (7,688 completed observations), whereas motivational conflicts and motivational interference were assessed in the second to sixth surveys of each day (5,274–5,275 completed observations). For day-level analyses examining perceived learning performance (assessed in the final survey of each day) 1,134 complete observations were available (1,428 scheduled assessments), corresponding to a compliance rate of 79.4%.

To examine temporal stability, rank-order stability was assessed separately for each MAQ subscale by calculating Pearson correlations between T0, T1, and T2. Ninety-five percent confidence intervals were reported for all correlations. Test–retest reliability was estimated using intraclass correlation coefficients (ICC) based on a two-way random-effects model with absolute agreement, for single measurements [ICC(2,1); Koo and Li, 2016]. Mean-level changes across time were analyzed using repeated-measures analyses of variance (ANOVA) for each subscale. Mauchly’s test was used to assess the assumption of sphericity. When this assumption was violated, Greenhouse–Geisser corrected results were reported. Effect sizes are presented as partial eta squared (*η^2^*).

### 3.2 Results of Study 2

#### 3.2.1 Descriptive statistics

Table 5 displays the means, standard deviations, and internal consistencies of the subscales of the MAQ-24, while descriptive statistics for all other study variables are reported in Appendix F. The subscales showed good to excellent internal consistency.

**Table 5:** Means, standard deviation and internal consistencies of the MAQ-24 (T0).

Questionnaire	Items	*M*	SD	α	95% CI	ω	Inter-item correlation
Differentiating motivation	8	3.89	0.78	0.82	[0.81, 0.82]	0.82	0.36
Verbal sharing of motivation	8	3.81	0.91	0.90	[0.90, 0.91]	0.90	0.54
Analyzing one’s own motivation	8	3.73	0.92	0.89	[0.88, 0.89]	0.89	0.49

MAQ-24 = Motivational Awareness Questionnaire; Items = Number of items, α = Cronbach’s alpha, CI = confidence interval; ω = McDonald’s omega.

The Descriptive Statistics of the variables from the ESM survey are displayed in Table 6.

**Table 6:** Descriptive statistics of the dependent variables.

Questionnaire	Number of observations	*N* = 102		*ICC*
		*M* (*SD*)	Min – Max	
PANAVA (ESM)				
Valence	7,688	0.96 (1.33)	−3 - 3	0.20
Positive	7,688	−0.90 (1.20)	−3 - 3	0.56
Negative	7,687	0.01 (1.30)	−3 - 3	0.38
Perceived Learning Performance	1,134	7.22 (3.98)	0–15	0.32
Motivational Conflicts-Should	5,275	0.19 (0.40)	0–1	0.21
Motivational Conflicts-Want	5,275	0.19 (0.36)	0–1	0.13
Motivational Interference	5,274	1.92 (0.85)	1–5	0.33

PANAVA = Short Scales for Assessing Positive Activation, Negative Activation, and Valence in Experience Sampling Studies.

#### 3.2.2 Psychometric properties of the MAQ scores

The MAQ-24 subscales showed moderate to strong intercorrelations at T0. *Differentiating motivation* correlated strongly with *verbal sharing of motivation* (*r* = 0.69, *p* < 0.01) but only weakly with *analyzing one’s own motivation* (*r* = 0.19). *Verbal sharing of motivation* and *analyzing one’s own motivation* were moderately correlated (*r* = 0.38, *p* < 0.01; *N* = 98–102).

To evaluate the factor structure of the MAQ, Bayesian CFAs were conducted on the 24 items of the three-factor model (*differentiating motivation*, *verbal sharing of motivation*, *analyzing one’s own motivation*) using a minimum of 100,000 MCMC iterations and a thinning factor of 10 to ensure chain independence. Standardized factor loadings ranged from 0.36 to 0.85, and all latent factors were significantly correlated (F1-F2 = 0.79, F1-F3 = 0.22, F2-F3 = 0.42). Model fit assessed via posterior predictive checking indicated a poor fit, with a posterior predictive *p*-value (*PPP*) of 0.000 and a 95% confidence interval for the difference between the observed and replicated chi-square values of [124.90, 249.10], which did not include zero. Consistent with the poor *PPP*-value, the Bayesian fit indices indicated a poor to marginal fit: *BCFI* = 0.830 [90% CI: 0.810, 0.846], *BTLI* = 0.813 [90% CI: 0.792, 0.831], and *BRMSEA* = 0.092 [90% CI: 0.088, 0.097]. The Bayesian model comparison indices for this model were *DIC* = 6832.42 and *BIC* = 7032.19. Good chain convergence (*PSR*/*R-hat* = 1.00) and high effective sample sizes were achieved across all parameters (lowest ESS = 2,921), confirming that the posterior parameter estimates were stable and highly reliable. All parameter-level estimates, including two-sided 95% confidence intervals, *R-hat* values, and effective sample sizes (*ESS*), are reported in Appendix G.

In comparison, the alternative one-factor model demonstrated a substantially worse and highly inadequate fit: *PPP* = 0.000 (with a 95% confidence interval for the difference between the observed and replicated chi-square values of [523.13, 1295.07]), *BCFI* = 0.186 [90% CI: 0.000, 0.546], *BTLI* = 0.634 [90% CI: 0.544, 0.796], and *BRMSEA* = 0.129 [90% CI: 0.096, 0.144]. The comparison of the information criteria (*DIC* = 7240.30 and *BIC* = 8278.34 for the one-factor model) further supported the superior fit of the three-factor structure over the one-factor model, despite the overall fit limitations of the three-factor model.

#### 3.2.3 Convergent associations

Table 7 presents the correlations between the MAQ-24 subscales and selected external criteria. Positive correlations indicate that greater *differentiating motivation*, *verbal sharing of motivation*, and *analyzing one’s own motivation* are associated with higher scores in related constructs such as emotional awareness, mindfulness facets, and self-control, whereas negative correlations were observed with measures of experienced motivational interference and motivational conflicts.

**Table 7:** Correlations between the MAQ-24 (T0) scales and the relevant constructs.

Questionnaire	Differentiating motivation	Verbal sharing of motivation	Analyzing one’s own motivation
TFL	0.28**	0.22*	0.04
EAQ			
EAQ-differentiating	0.54**	0.38**	−0.01
EAQ-verbal sharing	0.26**	0.33**	0.02
EAQ-analyzing emotion	0.24*	0.22*	0.43**
Self-control	0.31**	0.44**	0.34**
KIMS-D			
KIMS-D–observe	−0.05	0.02	0.23*
KIMS-D–attention	0.39**	0.34**	0.10
KIMS-D–describe	0.25*	0.32**	0.21*
KIMS-D–accept	0.12	0.20*	0.14
TMI			
TMI-S	−0.44**	−0.27**	−0.08
TMI-L	−0.47**	−0.22*	0.03
Motivational conflicts	−0.30**	−0.21*	0.00

EAQ = Emotion Awareness Questionnaire; KIMS-D = Kentucky Inventory of Mindfulness Skills; TMI = Tendency to Experience Motivational Interference during Studies (TMI-S) and during Leisure Time (TMI-L). *p < 0.05. **p < 0.01.

The results of the multilevel regression analyses and two-level logistic models examining whether trait motivational awareness (MAQ) predicted daily outcomes are presented in Table 8. Higher differentiation of motivation (MAQ_D) was significantly associated with lower negative affect (PAN_N; *B* = − 0.33, *SE* = 0.15, 95% CI [−0.62, −0.04], *p* = 0.025, *β* = −0.34) and with lower experience of motivational interference (MI; *B* = −0.26, *SE* = 0.10, 95% CI [−0.46, −0.07], *p* = 0.007, *β* = −0.40). Several additional associations did not reach statistical significance at the 0.05 level. No significant associations were observed for motivational conflicts (MC_W, MC_S) or perceived performance (all *p* > 0.25); these results are therefore not displayed in Table 8. For MC_W, MAQ_D (OR = 1.01, 95% CI [0.72, 1.43]), MAQ_V (OR = 0.87, 95% CI [0.66, 1.15]), and MAQ_A (OR = 1.05, 95% CI [0.88, 1.27]) were not significant predictors. Similarly, for MC_S, MAQ_D (OR = 1.03, 95% CI [0.64, 1.67]), MAQ_V (OR = 0.84, 95% CI [0.61, 1.17]), and MAQ_A (OR = 1.17, 95% CI [0.89, 1.53]) did not significantly predict conflict occurrence. A complete version of Table 8 including all predictors and outcomes is provided in Appendix H.

**Table 8:** Multilevel regression analyses of the average values of specific variables on the MAQ scales at T0.

Outcome	Predictor	B (unstandardized)	SE	*β* (STDYX)	*z*	95% CI (B)	*p*
PAN_P	MAQ_D	0.29	0.17	0.26	1.74	[−0.04, 0.61]	0.080
	MAQ_V	−0.03	0.12	−0.04	−0.27	[−0.26, 0.20]	0.791
	MAQ_A	0.02	0.08	0.03	0.26	[−0.14, 0.19]	0.792
PAN_N	MAQ_D	−0.33	0.15	−0.34	−2.25	[−0.62, −0.04]	0.025*
	MAQ_V	0.07	0.12	0.09	0.57	[−0.17, 0.31]	0.567
	MAQ_A	−0.01	0.07	−0.01	−0.10	[−0.15, 0.13]	0.919
PAN_V	MAQ_D	0.04	0.14	0.04	0.31	[−0.23, 0.31]	0.761
	MAQ_V	0.05	0.10	0.07	0.53	[−0.15, 0.25]	0.596
	MAQ_A	0.13	0.07	0.17	1.81	[−0.01, 0.27]	0.072
MI	MAQ_D	−0.26	0.10	−0.40	−2.69	[−0.46, −0.07]	0.007**
	MAQ_V	0.05	0.06	0.10	0.78	[−0.08, 0.17]	0.437
	MAQ_A	−0.02	0.06	−0.03	−0.28	[−0.13, 0.10]	0.777

PAN_P = positive affect, PAN_N = negative affect, PAN_V = valence, MI = experienced motivational interference, MAQ_D = differentiating motivation, MAQ_V = verbal sharing of motivation, MAQ_A = analyzing one’s own motivation. *p < 0.05. **p < 0.01.

To further examine the temporal stability of motivational awareness, additional analyses were conducted, and correlations and ICCs between the measurement points T0, T1, and T2 were calculated. Means and standard deviations for each subscale at T0, T1, and T2 are presented in Table 9. Missing repeated observations were handled using listwise deletion, resulting in varying sample sizes across time points (see Table 9). The high and significant correlations over time indicate a considerable degree of rank-order stability of the construct. These exploratory analyses were not corrected for multiple testing and should therefore be interpreted as hypothesis-generating.

**Table 9:** Means, standard deviations, correlations, and intraclass correlation coefficients of the MAQ across T0, T1, and T2.

Subscales	M (SD) T0	M (SD) T1	M (SD) T2	T0-T1 r [95% CI]	T1-T2 r [95% CI]	T0-T2 r [95% CI]	T0–T1 ICC [95% CI]	T1–T2 ICC [95% CI]	T0–T2 ICC [95% CI]
Differentiating motivation	3.90 (0.78)	4.16 (0.83)	4.16 (0.88)	0.56** [0.40, 0.69] (*N* = 90)	0.74** [0.62, 0.82] (*N* = 89)	0.49** [0.31, 0.63] (*N* = 92)	0.52 [0.33, 0.67]	0.73 [0.62, 0.82]	0.45 [0.26, 0.60]
Verbal sharing of motivation	3.81 (0.91)	4.08 (0.86)	4.10 (0.91)	0.45** [0.26, 0.56] (*N* = 88)	0.69** [0.56, 0.79] (*N* = 87)	0.48** [0.30, 0.63] (*N* = 90)	0.43 [0.24, 0.59]	0.69 [0.57, 0.79]	0.46 [0.28, 0.61]
Analyzing one’s own motivation	3.73 (0.92)	3.77 (0.91)	3.65 (0.98)	0.76** [0.65, 0.83] (*N* = 90)	0.81** [0.73, 0.87] (*N* = 88)	0.67** [0.54, 0.77] (*N* = 91)	0.76 [0.65, 0.83]	0.81 [0.72, 0.87]	0.67 [0.54, 0.77]

Confidence intervals are two-sided. Pearson correlation intervals based on Fisher’s r-to-z transformation; ICC = intraclass correlation coefficient, based on a two-way random-effects model with absolute agreement for single measurements (ICC(2,1)). ^⁎⁎^*p* < 0.01.

For the subscale *differentiating motivation*, Mauchly’s test indicated a violation of the assumption of sphericity, χ^2^ (2) = 9.20, *p* = 0.010. Therefore, Greenhouse–Geisser corrected results are reported. The effect of time was *F*(1.82, 160.00) = 9.26, *p* < 0.001, partial *η^2^* = 0.10. *Post hoc* pairwise comparisons (Bonferroni-corrected) indicated that scores were significantly higher at T1 than at T0 (Mean difference = 0.30, *p* < 0.001, 95% CI [0.11; 0.49]) and significantly higher at T2 than at T0 (Mean difference = 0.27, *p* = 0.007, 95% CI [0.06; 0.48]), while T1 and T2 did not differ significantly (Mean difference = 0.03, *p* = 1.00, 95% CI [− 0.13; 0.19]). Similarly, for the subscale *verbal sharing of motivation*, Mauchly’s test indicated that the assumption of sphericity was violated, χ^2^ (2) = 7.98, *p* = 0.018. Therefore, Greenhouse–Geisser corrected results are reported. The effect of time was *F*(1.83, 155.87) = 6.18, *p* = 0.003, partial *η^2^* = 0.07.

Similarly, *post hoc* pairwise comparisons (Bonferroni-corrected) for *verbal sharing of motivation* indicated that scores were significantly higher at T1 than at T0 (Mean difference = 0.28, *p* = 0.018, 95% CI [0.04; 0.52]) and significantly higher at T2 than at T0 (Mean difference = 0.28, *p* = 0.016, 95% CI [0.04; 0.51]), while T1 and T2 did not differ significantly (Mean difference = 0.00, *p* = 1.00, 95% CI [−0.18; 0.19]).

For the subscale *analyzing one’s own motivation*, Mauchly’s test likewise indicated a violation of sphericity, χ^2^ (2) = 10.802, *p* = 0.005, and Greenhouse–Geisser corrected results are therefore presented. The effect of time was not statistically significant, *F*(1.79, 155.63) = 1.48, *p* = 0.231, partial *η^2^* = 0.02. As the omnibus effect was not significant, no *post hoc* comparisons were conducted for this subscale.

### 3.3 Brief discussion

Although the CFA in Study 2 failed to confirm the revised three-factor structure due to poor model fit, some preliminary evidence for external associations emerged. The analyses showed that the subscale *differentiating motivation* was significantly associated with lower negative affect and lower levels of experienced motivational interference in daily life. However, the positive association between *differentiating motivation* and positive affect was not statistically significant. No other significant relationships emerged. In sum, while these initial correlations are promising, they must be interpreted with caution given the scale’s current structural instability. The findings tentatively suggest that motivational awareness exhibits rank-order stability over time, while certain facets show signs of systematic mean-level change. Consistent with this, ICC values indicated poor to moderate or good absolute stability, particularly between T1 and T2 and for *analyzing one’s own motivation*, where ICC and rank-order correlations converged closely; the lower ICCs between T0 and T1 for *differentiation of motivation* and *verbal sharing of motivation* align with the mean-level increase observed during this interval.

## 4 General discussion

The results of both studies extend previous research on metamotivation (Fujita et al., 2024a; Miele and Scholer, 2018) by providing an initial attempt to develop and examine an instrument for assessing individual differences in motivational awareness.

Whereas earlier work primarily described the processes involved in monitoring and controlling motivational states (Miele and Scholer, 2018), the present findings suggest that the conscious perception of motivational states–comparable to emotional awareness in the affective domain–may represent a distinguishable and potentially measurable facet. The MAQ is intended to assess individuals’ self-reported capacity to differentiate, verbalize, and analyze their own motivation.

The results of the factor analysis from Study 1 indicate that the originally proposed five-factor model of the MAQ could not be empirically confirmed. Although the individual scales demonstrated acceptable to good internal consistencies, the confirmatory factor analysis did not yield a satisfactory model fit. In particular, the subscales *bodily awareness of motivation* and *attending to others’ motivation* showed weak results, suggesting that these dimensions do not represent distinct aspects of motivational awareness.

Reducing the questionnaire to three core subscales–*differentiating motivation*, *verbal sharing of motivation*, and *analyzing one’s own motivation*–resulted in an improvement in model fit and clearer factor loadings in the revised version. However, the model’s fitness remained unsatisfactory. These three dimensions may reflect the cognitive and communicative processes that are potentially relevant for a reflective understanding of one’s own motivation. *Differentiating motivation* refers to the ability to distinguish between different motivational states; *verbal sharing of motivation* captures the tendency or perceived competence to express motivational states verbally; and *analyzing one’s own motivation* describes the reflection on the causes and consequences of one’s own motivational states. The findings from both studies provide preliminary indications for the convergent association of the Motivational Awareness Questionnaire (MAQ). Given the inadequate model fit and the heterogeneous relations of the subscales to external criteria, the interpretation of the MAQ total score appears unwarranted in the absence of support for a higher-order or bifactor structure. Therefore, the analyses reported above focused on the subscale level rather than on a total score.

Across the two samples, significant correlations emerged between the MAQ subscales and theoretically related constructs - mindfulness and the tendency to experience motivational interference in both studies, academic self-concept in Study 1, and emotion awareness in Study 2. These results support the assumption that motivational awareness is embedded within a broader network of self-regulatory and metacognitive processes.

In particular, *differentiating motivation* were both negatively correlated with motivational conflicts and with motivational interference, during studies as well as in leisure time. *Verbal sharing of motivation* showed the same pattern for motivational interference, whereas its negative association with motivational conflicts reached significance only in Study 2. This suggests that individuals who are better at differentiating and verbalizing their motivational states tend to perceive fewer conflicts between goals and report fewer distractions, feelings of frustration, or thoughts about alternative activities during an ongoing task. This pattern indicates that a more conscious access to one’s own motivation may be associated with better maintenance of task focus. A possible explanatory model is that individuals with a differentiated and reflective understanding of their motivation can recognize the emergence of motivational conflicts at an early stage and employ adaptive regulation strategies before motivational interference occurs.

Among the subscales, *differentiating motivation* appears to represent the most robust component of the construct, as it shows consistent associations with several relevant outcomes. In contrast, the dimensions *verbal sharing of motivation* and *analyzing one’s own motivation* seem to require further conceptual clarification and psychometric refinement.

The supplementary exploratory longitudinal analyses provide initial indications of the temporal stability of motivational awareness. The significant correlations between measurement time points suggest a moderate rank stability of the subscales. This suggests that individual differences tend to persist over the period under investigation and that the construct may, at least in part, comprise stable, dispositional components.

At the same time, the analyses of changes in mean scores indicate that not all facets are equally stable. For some subscales, changes were observed over time, while others showed no significant differences in mean scores. This pattern was further corroborated by the ICC values reported above, which showed lower absolute agreement specifically for the subscales *differentiating motivation* and *verbal sharing of motivation*. This could suggest that, while motivational awareness exhibits a certain degree of stability in its relative level of expression across individuals, individual components may be susceptible to change.

Overall, the findings tend to support a construct comprising both stable and potentially variable components. However, given the exploratory nature of the analyses and the limited duration of the study, these results should be interpreted with caution and need to be confirmed by further longitudinal studies.

A notable strength of this study is the methodological diversity, particularly the inclusion of situational-level data using ambulatory assessment. By combining trait data (questionnaire data) and state measures (experience sampling), the construct was examined at both the person and situation levels. Higher differentiation of motivation was associated with lower daily negative affect and reduced motivational interference. Although these effects were statistically significant, they were moderate in magnitude and should therefore be interpreted cautiously. The pattern suggests that individuals who report greater motivational differentiation also tend to report fewer distractions, feelings of frustration, or thoughts about alternative activities during ongoing goal pursuit, which may coincide with lower subjective emotional strain in everyday life.

At the same time, several hypothesized associations did not reach statistical significance. The absence of significant relationships with additional variables points to a differentiated positioning of motivational awareness within self-regulatory processes. *Differentiating motivation* appears to be less closely related to the occurrence of conflict situations per se than to their subjective processing. Whereas motivational conflicts in everyday life often arise situationally from external demands or competing goal constellations (Hofer and Fries, 2016), motivational awareness may instead be associated with how such situations are evaluated, interpreted, and emotionally experienced.

Taken together, the situational-level findings should be considered preliminary. While differentiation of motivation showed consistent associations with negative affect and motivational interference, many other hypothesized relationships were not supported. The present results therefore provide selective rather than comprehensive evidence for the role of motivational awareness in everyday self-regulation and warrant replication in future research, with larger samples.

Conceptually, the construct of motivational awareness may extend the scope of cognitive and affective self-regulation. Similar to emotional awareness (Rieffe et al., 2008), it encompasses the ability to perceive and interpret internal states, but focuses specifically on motivational domain. In line with our conceptualization, motivational awareness is understood as the perceptual prerequisite on which metamotivational monitoring and control can build, rather than as the monitoring process itself. While emotional awareness concerns the identification and differentiation of affective states (Rieffe et al., 2008) and metacognition refers to the monitoring of cognitive processes and learning strategies (Flavell, 1979), motivational awareness specifically targets the identification, differentiation, and analysis of motivational states. Whereas mindfulness emphasizes a non-judgmental present-moment awareness (Ströhle et al., 2010), motivational awareness involves a more analytic evaluation of one’s motivational drives and their underlying dynamics. Within the theoretical context of self-regulated learning, our findings suggest that successful self-regulation depends not only on knowledge of cognitive strategies but also to a significant extent on the ability to recognize, understand, and regulate one’s own motivational states (Miele and Scholer, 2018).

It can be assumed that students with higher motivational awareness are able to identify early signs of declining motivation and consequently apply regulatory strategies in a targeted manner. Thus, motivational awareness may be understood as a potentially relevant factor in metamotivational control and as a variable that could be associated with lower motivational interference in academic settings.

Furthermore, the moderate to strong correlations between the MAQ and related constructs such as emotional awareness, mindfulness and metacognition raise questions regarding its discriminant validity. Although these associations support convergent associations, they do not in themselves demonstrate that the MAQ captures a distinct construct rather than reflecting broader self-reflective tendencies.

Recent research points to potential conceptual links between emotional processes and metamotivational knowledge. Lee et al. (2026) demonstrated in a series of experimental studies that emotional experiences can function as informative cues from which individuals infer their current motivational states. These metamotivational inferences, in turn, guide strategic task selection–such as the preference for more “eager” (approach-oriented) versus “vigilant” (avoidance-oriented) tasks–in order to establish a fit between motivational state and task requirements (task–motivation fit).

These findings suggest a possible functional mechanism that may help explain the empirically observed relationships between emotional and metamotivational constructs. Importantly, however, such a functional linkage does not imply construct equivalence. Rather, emotional processes – or awareness of one’s own emotions – may represent one potential informational source that contributes to motivational awareness, while the underlying constructs remain theoretically distinguishable.

Nevertheless, more rigorous empirical tests are required to establish clearer construct boundaries. In particular, future research should examine discriminant validity using joint measurement models that include the MAQ and closely related constructs within the same confirmatory factor analytic framework. Given the relatively small sample sizes in the present studies, such integrated models would likely yield unstable parameter estimates and were therefore not conducted here. Larger and more diverse samples are needed to more definitively evaluate the distinctiveness of motivational awareness.

Beyond these statistical constraints, the generalizability of the findings is further limited by the specific sampling context. The relative homogeneity of the sample (predominantly recruited from one university with a high proportion of psychology students and predominantly female participants) restricts the generalizability of the findings to other disciplines and study programs. The predominance of participants with highly similar academic and demographic backgrounds may have limited the observed variability in motivational awareness, thereby restricting the convergent associations of our results. It remains to be seen whether the construct yields similar results outside the student population. Consequently, future research utilizing larger and more heterogeneous samples is needed to evaluate the robustness and generalizability of the MAQ.

Furthermore, although the MAQ demonstrated satisfactory psychometric properties in the present samples, we did not test for measurement invariance across gender, field of study, language, or cultural background. Therefore, it remains unclear whether the construct operates equivalently across these groups.

In addition, the relatively small sample sizes - particularly in the second study - present a limitation for conducting confirmatory factor analysis, as they can reduce the stability of parameter estimates and the interpretability of the fit indices. In Study 2, this sample size was primarily constrained by the highly demanding nature of the intensive longitudinal design (i.e., multiple daily prompts over 2 weeks), which naturally limits recruitment due to high participant burden. To address this limitation, we employed Bayesian estimation, which does not rely on large-sample asymptotic theory and provides credibility intervals that reflect parameter uncertainty. Nonetheless, this small sample likely introduced instability into the model. Therefore, the fit indices and specific parameter estimates must be interpreted with caution.

The conceptual orientation of some items warrants critical discussion. While a preference or desire to understand one’s own motivation is conceptually distinct from the actual ability to do so, self-report questionnaires frequently use habitual interests or tendencies as proxy indicators of awareness (e.g., Rieffe et al., 2008; Schwinger et al., 2007). Similarly, several items of the subscales *verbal sharing* and *analyzing one’s own motivation* may capture communicative confidence or willingness to share one’s motivation rather than motivational awareness itself. As the MAQ is a preliminary instrument, in future revisions, systematic expert assessment and cognitive interviewing remain necessary to achieve a clearer distinction between mere preferences and actual metacognitive monitoring. In particular, each item should be examined individually with regard to its conceptual alignment.

Moreover, the exclusive reliance on self-report data may also lead to biases. Future research should therefore aim to include objective indicators - such as behavioral measures, physiological data, or observer ratings - to further strengthen the construct validity. To enhance generalizability, a linguistic and cultural validation of the item formulations would also be advisable to ensure a consistent, context-independent understanding of the MAQ.

Despite these limitations, the studies provide an initial foundation for the further development and validation of the MAQ, as well as for future research on the role of motivational awareness in self-regulated learning. However, substantial further development is required before firm conclusions can be drawn. In particular, all items should be critically re-examined and tested in larger and more diverse samples to ensure their psychometric robustness.

Moreover, future research should investigate whether all subscales demonstrate comparable validity and stability, or whether certain components - such as the differentiation of one’s own motivation - represent particularly promising and robust facets of motivational awareness.

Thus, rather than representing a finalized instrument, the MAQ should currently be considered a developing measure that requires continued refinement and validation. In the long term, incorporating motivational awareness into theoretical models could help clarify the mechanisms through which learners actively regulate and sustain their motivation. This has important implications not only for basic research but also for the design of learning environments and interventions.

In a recent study, Hubley et al. (2025) examined how metamotivational knowledge regarding *task–motivation fit* differs between children (age range: 7.0–8.9 years) and adults. The results show that children already possess an emerging understanding that different tasks require different forms of motivation; however, this understanding is less sophisticated compared to that of adults. This suggests that metamotivational knowledge develops over time and can potentially be fostered through targeted support. Teo et al. (2025) also demonstrated in a recent study that high metamotivational knowledge among students significantly predicted their exam performance, and that metamotivational knowledge increased with students’ age. Moreover, there was no significant correlation between the size of the students’ strategy repertoire and their ability to accurately evaluate those strategies. Longitudinal and intervention studies would also be valuable to examine whether and to what extent metamotivational knowledge and in particular motivational awareness can be changed over time.

From a practical perspective, the MAQ represents a promising tool for research and developmental purposes, as it allows for the assessment of individual differences in how people deal with their motivational states. However, its use as a diagnostic instrument in counselling or training contexts would require further validation, including evidence for measurement invariance, normative data, and support for decision-making at the individual level. Future research should therefore examine these aspects before practical diagnostic applications can be recommended.

In summary, given the inconsistent model fit, further psychometric improvement and cross-validation are required before firm conclusions can be drawn. Nevertheless, the construct of motivational awareness provides an initial step toward understanding how individuals perceive and regulate their own motivation, and the present findings offer preliminary support for its conceptual relevance, although further research is needed to clarify the mechanisms linking motivational awareness to self-regulated learning and motivational regulation.

It may serve as a useful starting point for investigating individual differences in motivational self-knowledge and for stimulating future research in the areas of learning, metamotivation, and self-regulation.

## Data Availability

The raw data supporting the conclusions of this article will be made available by the authors, without undue reservation.
