# Supplementary material for: First steps toward a questionnaire of motivational awareness: preliminary development and psychometric examination
Source: Front Psychol. 2026 Aug 31;17:1869033. doi: 10.3389/fpsyg.2026.1869033 (PMC13572709; doi:10.3389/fpsyg.2026.1869033)
Supplement: Supplementary file 1 [file Supplementary_file_1.pdf]

6 Appendix

Appendix A

MAQ items (28 items)

Differentiating Motivation

1.1 I am often confused or at a loss as to whether I am motivated or unmotivated. -R

1.2 I often can't say what motivates me. -R

1.3 I often can't say why I do or don't do something. -R

1.4 I find it difficult to say whether I am motivated or unmotivated. -R

1.5 I often don't know exactly whether I do something because I like doing it or because I feel obliged to. -R

1.6 I usually know what motivates me.

1.7 I know what I like to do.

1.8 When I am unmotivated, I do not know what feeling (e.g., fear, boredom, hopelessness) could be responsible. -R

1.9 Sometimes I feel unmotivated and don't know why. -R

Verbal Sharing of Motivation

2.1 I find it difficult to explain to a friend what motivates me. -R

2.2 I find it difficult to talk to someone about my motivation. -R

2.3 I can easily explain to a friend how I motivate myself.

2.4 I can easily explain to a friend why I am unmotivated.

Bodily Awareness

3.1 When I am unmotivated, my body feels exhausted.

3.2 When I am unmotivated, my body feels tired and sluggish.

3.3 When I am motivated, my body feels energized.

3.4 I notice a physical change depending on whether I am motivated or unmotivated.

Attending to Others' Motivation

4.1 I have hardly any interest in what motivates my friends. -R

4.2 When a friend is unmotivated, I try to understand why.

4.3 I don't care how my friends motivate themselves. -R

4.4 I can usually tell whether my friends are motivated or unmotivated.

Analyzing one's own Motivation

5.1 When I am unmotivated, I try to understand why.

5.2 The nature of my motivation helps me to understand what I like to do.

5.3 If I have a motivation problem, it helps me to find out whether an emotion (e.g., fear, hopelessness, boredom) could be responsible.
5.4 It is important for me to understand whether I am motivated or unmotivated.
5.5 I always want to know how I can motivate myself.
5.6 I know exactly how to motivate myself.
5.7 When I am unmotivated, I try to analyze how I can motivate myself.

Note. Items marked with R are reverse-scored.

Appendix B

German Version MAQ items (28 items)

Differenzieren von Motivation

1.1 Ich bin oft verwirrt oder ratlos darüber, ob ich motiviert oder unmotiviert bin. -R

1.2 Ich kann oft nicht sagen, was mich antreibt. -R

1.3 Ich kann oft nicht sagen, warum ich etwas mache oder nicht mache. -R

1.4 Es fällt mir schwer zu sagen, ob ich motiviert oder unmotiviert bin. -R

1.5 Ich weiß oft nicht genau, ob ich etwas mache, weil ich es gerne mache, oder weil ich mich verpflichtet fühle. -R

1.6 Ich weiß in der Regel, was mich antreibt.

1.7 Ich weiß, was ich gerne mache.

1.8 Wenn ich unmotiviert bin, weiß ich nicht, welches Gefühl (z.B. Angst, Langeweile, Hoffnungslosigkeit) dafür verantwortlich sein könnte. -R

1.9 Manchmal fühle ich mich unmotiviert und weiß nicht warum. -R

Verbaler Austausch von Motivation

2.1 Ich finde es schwierig, einem:einer Freund:in zu erklären, was mich motiviert -R

2.2 Ich finde es schwierig mit jemandem über meine Motivation zu sprechen. -R

2.3 Ich kann einem:eine Freund:in leicht erklären, wie ich mich motiviere.

2.4 Ich kann einem:einer Freund/ einer Freundin leicht erklären, warum ich unmotiviert bin.

Körperliches Bewusstsein

3.1 Wenn ich unmotiviert bin, fühlt sich mein Körper erschöpft an.

3.2 Wenn ich unmotiviert bin, fühlt sich mein Körper müde und träge an.

3.3 Wenn ich motiviert bin, fühlt sich mein Körper energiegeladen an.

3.4 Ich bemerke eine körperliche Veränderung, je nachdem, ob ich motiviert oder unmotiviert bin.

Auf die Motivation anderer eingehen

4.1. Ich habe wenig Interesse daran, was meine Freund:innen motiviert. -R

4.2. Wenn ein:eine Freund:in unmotiviert ist, versuche ich zu verstehen warum.

4.3. Es ist mir egal, wie sich meine Freund:innen motivieren. -R

4.4. Ich erkenne meistens, ob meine Freund:innen motiviert oder unmotiviert sind.

Analyse von Motivation

5.1 Wenn ich unmotiviert bin, versuche ich zu verstehen, warum.

5.2 Die Art meiner Motivation hilft mir, zu verstehen, was ich gerne mache.

5.3 Wenn ich ein Motivationsproblem habe, hilft es mir herauszufinden, ob ein Gefühl (z.B. Angst, Hoffnungslosigkeit, Langeweile) dafür verantwortlich sein könnte.
5.4 Es ist mir wichtig zu verstehen, ob ich motiviert oder unmotiviert bin.
5.5 Ich will immer wissen, wie ich mich motivieren kann.
5.6 Ich weiß genau, wie ich mich motivieren kann.
5.7 Wenn ich unmotiviert bin, versuche ich zu analysieren, wie ich mich motivieren kann.

Note. Items marked with R are reverse-scored.

Appendix C

Means, Standard Deviation and Internal Consistencies of the TFL, Academic Success, SSI, KIMS-D, Academic Self-concept, TMI and Motivational Conflicts

**Questionnaire**	**Items**	**M**	**SD**	***α***	**Inter-Item Correlation**
TFL	4	3.04	.59	.87	.63
Academic Success	1	3.44	.92	-	-
SSI	15	2.46	.49	.89	.36
SSI – Motivation Control	5	2.43	.56	.80	.45
SSI- Attention Control	5	2.65	.59	.81	.46
SSI – Mood Management	5	2.30	.57	.79	.44
KIMS-D	39	3.25	.37	.84	.15
KIMS-D Observation	12	3.44	.59	.83	.29
KIMS-D Attention	10	2.94	.51	.74	.23
KIMS-D Describing	8	3.45	.73	.88	.48
KIMS-D Accepting	9	3.18	.60	.72	.20
Academic Self-Concept	5	4.37	.82	.77	.44
Motivational Conflicts	1	3.70	.98	-	-
TMI	22	2.87	.52	.89	.27
TMI-S	12	3.15	.61	.89	.40
TMI-L	10	2.54	.74	.92	.55

Note. TFL = Temporal facets of life satisfaction, Academic Success; SSI = Self-control Inventory; KIMS-D = Kentucky Inventory of Mindfulness Skills; Academic Self-concept; TMI = Tendency to Experience Motivational Interference during Studies (TMI-S) and during Leisure Time (TMI-L); Motivational Conflicts, Items = Number of items, α = Cronbach's alpha.

Appendix D

Deviations from Preregistration

Study	Preregistered Plan	Actual Procedure	Reason for Deviation
**Study 1**	**Only Original CFA with all items**	**Second CFA with removal of Items 1.6, 1.7, 5.6**	**Post hoc model refinement to improve fit**
**Study 2**	**Measure of general ability to control motivation, attention, and mood management**	**Self-Control Scale – Short Version (SCS-K-D; Bertrams & Dickhäuser, 2009) administered**	**Administrative oversight during survey preparation**
**Study 2**	**Conduct exploratory (principal axis) and confirmatory factor analyses**	**Only confirmatory factor analysis (CFA) conducted**	**The factor structure was theoretically specified a priori; focus placed on confirmatory testing**
**Study 2**	**No test–retest or stability analysis specified**	**Exploratory analysis of temporal stability conducted**	**Added post hoc to examine trait stability**
**Study 1 and 2**	**Estimate reliability using Cronbach's alpha**	**Estimated both Cronbach's alpha and McDonald's omega**	**To provide a more robust and precise estimate of internal consistency**

Appendix E

German Version MAQ items (24 items)

Differenzieren von Motivation

1.1 Ich bin oft verwirrt oder ratlos darüber, ob ich motiviert oder unmotiviert bin. -R

1.2 Ich kann oft nicht sagen, was mich antreibt.-R

1.3 Ich kann oft nicht sagen, warum ich etwas mache oder nicht mache.-R

1.4 Es fällt mir schwer zu sagen, ob ich motiviert oder unmotiviert bin. -R

1.5 Ich weiß oft nicht genau, ob ich etwas mache, weil ich es gerne mache, oder weil ich mich verpflichtet fühle. -R

1.6 Wenn ich unmotiviert bin, weiß ich nicht, welches Gefühl (z.B. Angst, Langeweile, Hoffnungslosigkeit) dafür verantwortlich sein könnte. -R

1.7 Manchmal fühle ich mich unmotiviert und weiß nicht warum. -R

1.8 Ich finde es leicht zu erkennen ob ich etwas freiwillig mache oder weil andere es von mir erwarten.

Verbaler Austausch von Motivation

2.1 Ich finde es schwierig, einem:einer Freund:in zu erklären, was mich motiviert. -R

2.2 Ich finde es schwierig mit jemandem über meine Motivation zu sprechen. -R

2.3 Ich kann einem:einer Freund:in leicht erklären, wie ich mich motiviere.

2.4 Ich kann einem:einer Freund:in leicht erklären, warum ich unmotiviert bin.

2.5 Ich kann gut beschreiben, was mich in bestimmten Situationen antreibt.

2.6 Es gelingt mir gut, meinen Motivationszustand verständlich zu beschreiben.

2.7 Es ist manchmal schwer für mich, jemandem zu erklären, warum ich motiviert oder unmotiviert bin. -R

2.8 Wenn ich über meine Motivation spreche, kann ich sie genau erklären.

Analyse von Motivation

3.1 Wenn ich unmotiviert bin, versuche ich zu verstehen, warum.

3.2 Meine Motivation hilft mir, zu verstehen, was ich gerne mache.

3.3 Wenn ich ein Motivationsproblem habe, hilft es mir herauszufinden, ob ein Gefühl (z.B. Angst, Hoffnungslosigkeit, Langeweile) dafür verantwortlich sein könnte.

3.4 Es ist mir wichtig zu verstehen, ob ich motiviert oder unmotiviert bin.

3.5 Ich will immer wissen, wie ich mich motivieren kann.

3.6 Wenn ich unmotiviert bin, versuche ich zu verstehen, wie ich mich motivieren kann.

3.7 Ich analysiere oft die Zusammenhänge zwischen meinen Emotionen und meiner Motivation.

3.8 Es ist mir wichtig, die Ursachen meines Motivationszustandes genau zu verstehen.

Note. Items marked with R are reverse-scored.

Appendix F

Means, Standard Deviation and Internal consistencies of the SCS-K-D; KIMS-D; TMI; EAQ; Motivational Conflicts, Motivation Interference

Questionnaire	Items	M	SD	*α*	Inter-Item Correlation
KIMS-D					

Questionnaire	Items	M	SD	α	Inter-Item Correlation
KIMS-D Observe	12	3.27	.57	.78	.23
KIMS-D Attention	10	2.84	.59	.81	.31
KIMS-D Describe	8	3.41	.80	.89	.49
KIMS-D Accept	9	3.23	.60	.66	.17
TMI					
TMI-S	12	3.67	.76	.87	.37
TMI-L	10	2.59	.76	.92	.55
EAQ					
EAQ Differentiating	7	4.13	.69	.62	.18
EAQ Verbal Sharing	3	3.93	1.34	.88	.72
EAQ Analyzing	5	3.73	.917	.81	.48
Self-Control	13	3.05	.60	.81	.24
Motivational Conflicts	1	3.60	.89	-	-

Note. MAQ-24 = Motivational Awareness Questionnaire; KIMS-D = Kentucky Inventory of Mindfulness Skills; TMI = Tendency to Experience Motivational Interference during Studies (TMI-S) and during Leisure Time (TMI-L); Motivational Conflicts; EAQ= Emotion Awareness Questionnaire; Self-control = Self-control Inventory; Items = Number of items, α = Cronbach's alpha.

Appendix G

Parameter-Level Posterior Estimates, Standard Deviations, 95% Confidence Intervals, R-hat, and Effective Sample Size for the Bayesian Three-Factor CFA (Time 0)

Parameter	Estimate	SD	95% CI	β (STDYX)	95% CI	R-hat	ESS
Factor Loadings on F1 (Differentiating Motivation)							

Parameter	Estimate	SD	95% CI	β (STDYX)	95% CI	R-hat	ESS
**D_01_R (marker item, fixed = 1)**	1.00	-	-	0.66	[0.49, 0.78]		
**D_02_R**	1.08	0.22	[0.76, 1.59]	0.68	[0.53, 0.78]	1.00	5515
**D_03_R**	1.20	0.23	[0.86, 1.73]	0.73	[0.59, 0.83]	1.00	5059
**D_04_R**	1.20	0.24	[0.85, 1.77]	0.71	[0.57, 0.81]	1.00	5072
**D_05_R**	0.99	0.23	[0.61, 1.53]	0.53	[0.35, 0.68]	1.00	6961
**D_06_R**	0.95	0.22	[0.60, 1.48]	0.55	[0.38, 0.69]	1.00	6782
**D_07_R**	1.06	0.24	[0.70, 1.62]	0.62	[0.46, 0.74]	1.00	5464
**D_08**	0.62	0.21	[0.25, 1.08]	0.36	[0.15, 0.53]	1.00	9720
Factor Loadings on F2 (Verbal Sharing of Motivation)							
**V_01_R (marker item, fixed = 1)**	1.00	-	-	0.80	[0.69, 0.87]		
**V_02_R**	0.92	0.14	[0.68, 1.21]	0.68	[0.54, 0.78]	1.00	12792
**V_03**	1.03	0.12	[0.83, 1.30]	0.85	[0.77, 0.90]	1.00	9998
**V_04**	0.97	0.12	[0.76, 1.25]	0.79	[0.69, 0.86]	1.00	10712
**V_05**	0.67	0.11	[0.45, 0.92]	0.62	[0.46, 0.73]	1.00	13550
**V_06**	0.87	0.12	[0.66, 1.13]	0.72	[0.59, 0.81]	1.00	12090
**V_07_R**	0.97	0.12	[0.76, 1.24]	0.77	[0.66, 0.85]	1.00	11606
**V_08**	0.87	0.13	[0.65, 1.14]	0.71	[0.58, 0.81]	1.00	11909
Factor Loadings on F3 (Analyzing one's own Motivation)							

Parameter	Estimate	SD	95% CI	β (STDYX)	95% CI	R-hat	ESS
**A_01 (marker item, fixed = 1)**	1.00	-	-	0.84	[0.74, 0.90]		
**A_02**	0.56	0.10	[0.37, 0.77]	0.55	[0.38, 0.69]	1.00	17728
**A_03**	0.84	0.13	[0.61, 1.11]	0.64	[0.50, 0.76]	1.00	16239
**A_04**	0.93	0.11	[0.73, 1.17]	0.82	[0.72, 0.89]	1.00	12719
**A_05**	0.69	0.11	[0.49, 0.92]	0.64	[0.49, 0.75]	1.00	16164
**A_06**	0.75	0.11	[0.56, 0.97]	0.68	[0.53, 0.78]	1.00	17277
**A_07**	0.93	0.13	[0.69, 1.21]	0.70	[0.56, 0.80]	1.00	13409
**A_08**	0.93	0.12	[0.72, 1.19]	0.79	[0.68, 0.87]	1.00	12939
Factor Variances and Covariances							
**Variance F1**	0.51	0.16	[0.25, 0.88]			1.00	5764
**Variance F2**	0.96	0.22	[0.60, 1.47]			1.00	11295
**Variance F3**	1.16	0.25	[0.75, 1.75]			1.00	13559
**Covariance F1 WITH F2**	0.54	0.13	[0.33, 0.86]	0.79	[0.66, 0.88]	1.00	8874
**Covariance F1 WITH F3**	0.16	0.10	[-0.01, 0.38]	0.22	[-0.01, 0.43]	1.00	16669
**Covariance F2 WITH F3**	0.43	0.14	[0.20, 0.75]	0.42	[0.21, 0.58]	1.00	17601
Residual Variances							
**D_01_R**	0.66	0.12	[0.48, 0.93]			1.00	13700
**D_02_R**	0.71	0.12	[0.52, 0.98]			1.00	19918

Parameter	Estimate	SD	95% CI	β (STDYX)	95% CI	R-hat	ESS
**D_03_R**	0.64	0.12	[0.46, 0.91]			1.00	18990
**D_04_R**	0.72	0.13	[0.52, 1.01]			1.00	20000
**D_05_R**	1.27	0.20	[0.95, 1.73]			1.00	19100
**D_06_R**	1.04	0.16	[0.78, 1.42]			1.00	19893
**D_07_R**	0.92	0.15	[0.67, 1.27]			1.00	19309
**D_08**	1.32	0.20	[1.00, 1.79]			1.00	20000
**V_01_R**	0.56	0.10	[0.40, 0.79]			1.00	17296
**V_02_R**	0.95	0.15	[0.70, 1.30]			1.00	20000
**V_03**	0.40	0.08	[0.28, 0.58]			1.00	19510
**V_04**	0.55	0.09	[0.40, 0.76]			1.00	19933
**V_05**	0.72	0.11	[0.54, 0.97]			1.00	20000
**V_06**	0.67	0.11	[0.49, 0.93]			1.00	19326
**V_07_R**	0.63	0.11	[0.46, 0.87]			1.00	19391
**V_08**	0.71	0.12	[0.52, 0.98]			1.00	19768
**A_01**	0.50	0.11	[0.32, 0.74]			1.00	15863
**A_02**	0.84	0.13	[0.63, 1.14]			1.00	19917
**A_03**	1.15	0.18	[0.86, 1.56]			1.00	20000
**A_04**	0.48	0.10	[0.32, 0.71]			1.00	18525
**A_05**	0.81	0.13	[0.60, 1.10]			1.00	20000

Parameter	Estimate	SD	95% CI	β (STDYX)	95% CI	R-hat	ESS
**A_06**	0.77	0.13	[0.56, 1.08]			1.00	18396
**A_07**	1.06	0.18	[0.79, 1.47]			1.00	20000
**A_08**	0.60	0.12	[0.41, 0.86]			1.00	18345
Intercepts							
**D_01_R**	4.55	0.11	[4.34, 4.76]			1.00	10170
**D_02_R**	3.93	0.11	[3.71, 4.15]			1.00	10145
**D_03_R**	4.01	0.12	[3.78, 4.24]			1.00	9681
**D_04_R**	4.41	0.12	[4.18, 4.65]			1.00	9430
**D_05_R**	3.40	0.13	[3.14, 3.66]			1.00	12815
**D_06_R**	3.76	0.12	[3.52, 4.01]			1.00	12129
**D_07_R**	3.22	0.12	[2.98, 3.47]			1.00	11104
**D_08**	3.82	0.12	[3.58, 4.07]			1.00	16269
**V_01_R**	3.89	0.12	[3.65, 4.13]			1.00	7213
**V_02_R**	4.09	0.13	[3.83, 4.34]			1.00	8820
**V_03**	3.56	0.12	[3.32, 3.79]			1.00	6612
**V_04**	3.76	0.12	[3.52, 3.99]			1.00	7304
**V_05**	4.01	0.11	[3.80, 4.22]			1.00	10285
**V_06**	3.72	0.12	[3.51, 3.97]			1.00	8176
**V_07_R**	3.82	0.12	[3.58, 4.07]			1.00	7323

Parameter	Estimate	SD	95% CI	β (STDYX)	95% CI	R-hat	ESS
**V_08**	3.66	0.12	[3.42, 3.90]			1.00	8514
**A_01**	3.69	0.13	[3.43, 3.94]			1.00	8724
**A_02**	4.16	0.11	[3.93, 4.37]			1.00	13302
**A_03**	3.43	0.14	[3.16, 3.71]			1.00	11114
**A_04**	3.57	0.12	[3.33, 3.80]			1.00	8645
**A_05**	3.96	0.12	[3.73, 4.19]			1.00	11578
**A_06**	3.99	0.12	[3.75, 4.23]			1.00	10624
**A_07**	3.39	0.14	[3.10, 3.66]			1.00	10395
**A_08**	3.65	0.13	[3.40, 3.90]			1.00	9159

Note. N = 102*; CI* = confidence interval; *β* (STDYX) = fully standardized loading or factor correlation; *R-hat* = Gelman-Rubin potential scale reduction factor; *ESS* = effective sample size. Estimation used two MCMC chains with a maximum of 100,000 iterations (thinning = 10); the 10,000 post-convergence iterations per chain (factor score imputation iterations excluded) were used to compute R-hat and *ESS*.

Appendix H

Multilevel regression analyses of the average values of specific variables on the MAQ scales at T0

Outcome	Predictor	B (unstandardized)	SE	*β* (STDYX)	z	95% CI (B)	*p*
**PAN_P**	**MAQ_D**	**0.29**	**0.17**	**0.26**	**1.74**	**[- 0.04, 0.61]**	**.080**
	**MAQ_V**	**- 0.03**	**0.12**	**- 0.04**	**- 0.27**	**[- 0.26, 0.20]**	**.791**
	**MAQ_A**	**0.02**	**0.08**	**0.03**	**0.26**	**[- 0.14, 0.19]**	**.792**
**PAN_N**	**MAQ_D**	**- 0.33**	**0.15**	**- 0.34**	**- 2.25**	**[- 0.62, - 0.04]**	**.025***
	**MAQ_V**	**0.07**	**0.12**	**0.09**	**0.57**	**[- 0.17, 0.31]**	**.567**
	**MAQ_A**	**- 0.01**	**0.07**	**- 0.01**	**-0.10**	**[- 0.15, 0.13]**	**.919**

**PAN_V**	**MAQ_D**	**0.04**	**0.14**	**0.04**	**0.31**	**[- 0.23, 0.31]**	**.761**
	**MAQ_V**	**0.05**	**0.10**	**0.07**	**0.53**	**[- 0.15, 0.25]**	**.596**
	**MAQ_A**	**0.13**	**0.07**	**0.17**	**1.81**	**[- 0.01, 0.27]**	**.072**
**MC_W**	**MAQ_D**	**0.01**	**0.18**	**0.01**	**0.08**	**[- 0.33, 0.36]**	**.940**
	**MAQ_V**	**- 0.14**	**0.14**	**- 0.18**	**- 0.98**	**[- 0.42, 0.14]**	**.325**
	**MAQ_A**	**0.05**	**0.09**	**0.07**	**0.58**	**[- 0.13, 0.24]**	**.565**
**MC_S**	**MAQ_D**	**0.03**	**0.25**	**0.03**	**0.13**	**[- 0.45, 0.51]**	**.897**
	**MAQ_V**	**- 0.17**	**0.17**	**- 0.16**	**- 1.04**	**[- 0.50, 0.15]**	**.297**
	**MAQ_A**	**0.15**	**0.14**	**0.14**	**1.11**	**[- 0.12, 0.42]**	**.269**
**MI**	**MAQ_D**	**- 0.26**	**0.10**	**- 0.40**	**- 2.69**	**[- 0.46, - 0.07]**	**.007****
	**MAQ_V**	**0.05**	**0.06**	**0.09**	**0.78**	**[- 0.08, 0.17]**	**.437**
	**MAQ_A**	**- 0.02**	**0.06**	**- 0.03**	**- 0.28**	**[- 0.13, 0.10]**	**.777**
**Performance**	**MAQ_D**	**0.41**	**0.57**	**0.14**	**0.72**	**[- 0.70, 1.52]**	**.470**
	**MAQ_V**	**0.26**	**0.41**	**0.10**	**0.63**	**[- 0.44, 1.05]**	**.530**
	**MAQ_A**	**- 0.22**	**0.33**	**- 0.09**	**- 0.66**	**[- 0.34, 0.17]**	**.511**

Note. PAN_P = positive affect, PAN_N = negative affect, PAN_V = valence, MC_W = motivational want conflict, MC_S = motivational should conflict, MI= experienced motivational interference, Performance = perceived learning performance, MAQ_D = differentiating motivation, MAQ_V = verbal sharing of motivation, MAQ_A = analyzing one's own motivation, For MC_W and MC_S, coefficients are log-odds from two-level logistic models; odds ratios are reported in the text.
*p < .05. ** *p <.01*.
